# On the interplay between neoclassical tearing modes and nonlocal transport in toroidal plasmas

**DOI:** 10.1038/srep32697

**Published:** 2016-09-06

**Authors:** X. Q. Ji, Y. Xu, C. Hidalgo, P. H. Diamond, Yi Liu, O. Pan, Z. B. Shi, D. L. Yu

**Affiliations:** 1 Southwestern Institute of Physics, PO Box 432, Chengdu 610041, Peoples Republic of China; 2 Laboratorio Nacional de Fusión, CIEMAT, 28040 Madrid, Spain; 3 CMTFO and CASS, UCSD, La Jolla, California 92093, USA

## Abstract

This Letter presents the first observation on the interplay between nonlocal transport and neoclassical tearing modes (NTMs) during transient nonlocal heat transport events in the HL-2A tokamak. The nonlocality is triggered by edge cooling and large-scale, inward propagating avalanches. These lead to a locally enhanced pressure gradient at the *q* = 3/2 (or 2/1) rational surface and hence the onset of the NTM in relatively low *β* plasmas (*β*_*N*_ < 1). The NTM, in return, regulates the nonlocal transport by truncation of avalanches by local sheared toroidal flows which develop near the magnetic island. These findings have direct implications for understanding the dynamic interaction between turbulence and large-scale mode structures in fusion plasmas.

Over the past years, a number of experiments which study transient transport events in magnetically confined plasmas have revealed a provocative phenomenon^1^,^2^,^3^,^4^,^5^,^6^,^7^. A rapid change of local thermal transport in response to a change of plasma parameters occurs at a location distant from the perturbation, i. e., the so-called “nonlocal” transport, which cannot be explained just by the standard diffusive local transport paradigm. Although several theoretical models^8^,^9^,^10^,^11^,^12^ have been proposed which try to interpret this nonlocal effect, the underlying physical mechanisms remain unclear, due to limited experimental data.

On the other hand, to achieve economic viability for a future fusion reactor, a crucial task is to operate the plasma at relatively high *β* values, where *β* is the ratio of plasma pressure *p* to the magnetic field (*B*) pressure defined as *β* = *p*/(*B*^2^/2*μ*_0_). However, the maximum achievable *β* can be limited by resistive MHD instabilities, in particular, the neoclassical tearing mode (NTM)^13^,^14^,^15^,^16^,^17^. The NTM is driven by a loss of a large bootstrap current related to a large plasma pressure gradient within a magnetic island. The occurrence of NTMs may considerably increase parallel heat transport across the width of the island, degrade energy confinement and even cause plasma disruptions. Thus, the NTM will prevent plasmas moving into a high-*β* state if it appears in the low-*β* case. As a consequence, understanding the physical processes for the onset of NTMs, as well as their prevention, is also one of major challenges for fusion.

In this Letter, we present the first experimental observation of self-regulation of nonlocal transport events by NTMs generated during transient nonlocal transport events. The nonlocal effect is excited by edge cooling and propagates inward by avalanche events. These cause a local increase of the pressure gradient at the inversion surface, and thus the onset of the NTM in relatively low *β* plasmas (*β*_*N*_ < 1). The presence of the NTM, results in the development of sheared flows at the magnetic island. The dual role of low-order rationals as both damping^18^ and drive mechanism of steady-state^19^ and fluctuating^20^ *E*_*r*_ × *B* flows has been identified, thus the magnetic topology is an important regulator of radial electric fields, MHD activities and transport levels. These then truncate the nonlocal transport because of suppression of avalanches by shearing. A sketch of the dynamic flow chart is shown in Fig. 1. These results may have important implications for the understanding of multi-scale transport dynamics, i. e., the intimate interplay between small and large scale structures.

## Experimental Setup

The experiments were performed in deuterium L-mode plasmas at the HL-2A tokamak^21^ (*R* = 1.65 m, *a* = 0.4 m) heated by electron cyclotron resonant heating (ECRH) in both limiter and single-null divertor configurations. In this study, the interplay between nonlocal transport and the NTM was investigated in about 14 discharges. The maximal ECRH power can reach up to 3 MW. The plasma parameters are the plasma current *I*_*p*_ = 150–160 kA, toroidal magnetic field *B*_*T*_ = 1.28–1.33 T, line-averaged electron density 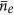 = (0.8–2.0) × 10^19^ m^−3^ and edge safety factor *q*_95_ ≈ 4. The nonlocal thermal transport events were triggered by employing a supersonic molecular beam injection (SMBI) or gas-puffing to generate cold pulses at the plasma boundary^5^. The equilibrium electron temperature (*T*_*e*_) profile and temperature fluctuations (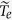) were measured by a 16 channel electron cyclotron emission (ECE) radiometer^22^ with 1.25 MHz digitizer. Besides, Mirnov coils, soft-x-ray (SXR) detectors and Doppler reflectometer were used to measure MHD instabilities and turbulence at a sampling rate of 1 MHz. The toroidal plasma rotation was detected by a charge exchange recombination spectroscopy (CXRS) with a spatial resolution ≈1.5 cm^23^.

## Experimental Results and Discussion

**Figure 2:**
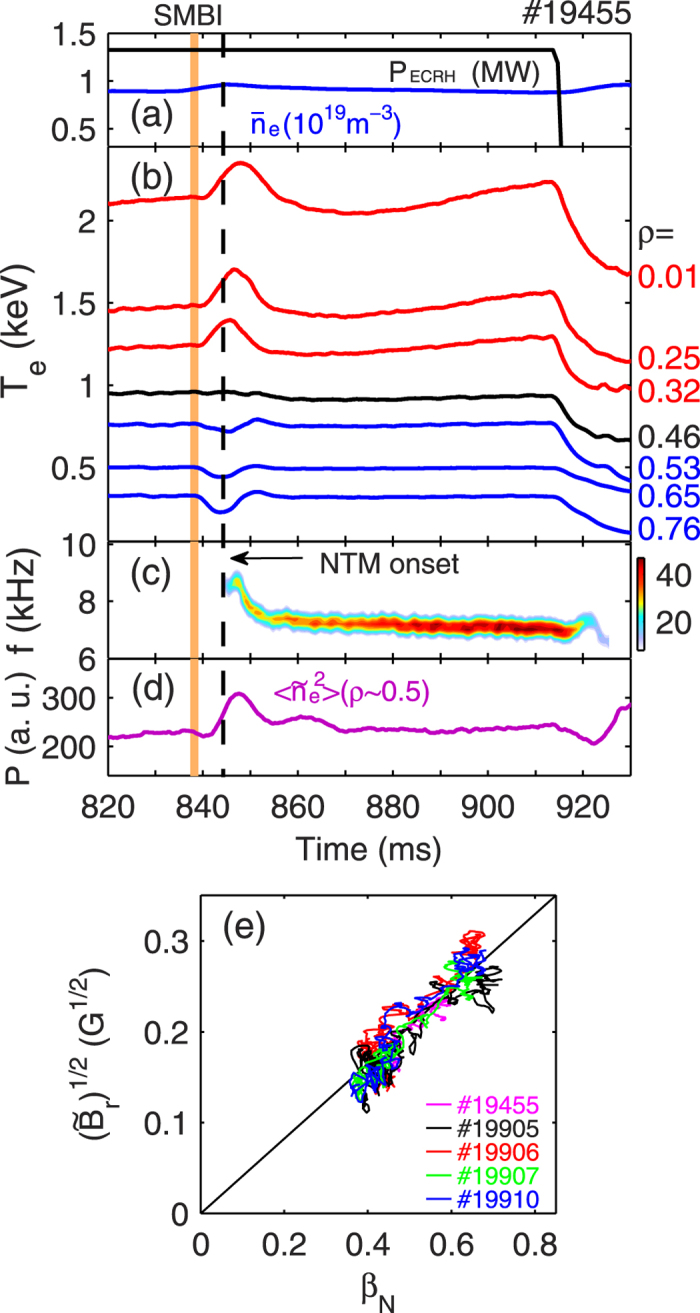
Typical discharge waveforms with an onset of a 3/2 NTM during the nonlocal transport induced by SMBI (vertical yellow bar) in ECRH-heated plasmas at HL-2A. (**a**) The line-averaged density and ECRH power; (**b**) multi-channel ECE signals showing increase of *T*_*e*_ in the plasma core (red) and decrease of *T*_*e*_ in the plasma edge (blue); (**c**) contour-plot of frequency spectrum of the SXR signal showing the onset of a 3/2 NTM (*f* ≈ 7 kHz) during the nonlocal phase; (**d**) the mean power of density fluctuations measured by a reflectometer at *ρ* ≈ 0.5; (**e**) variation of the square root of 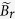 of the 3/2 island versus *β*_*N*_ in five discharges.

**Figure 3:**
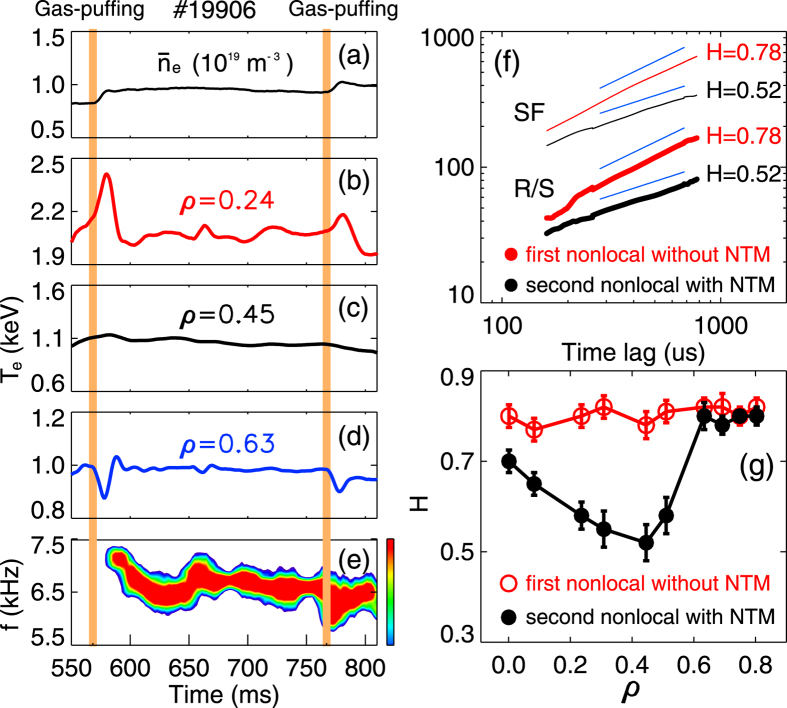
Impact of NTMs on the magnitude of nonlocal transport. (**a**–**e**) are time evolutions of plasma density, multi-channel ECE signals and contour-plot of frequency spectrum in the Mirnov signal showing reduction of nonlocal effects with the presence of the 3/2 NTM; (**f**) Hurst exponents calculated by SF and R/S methods in 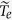 during two nonlocal phases at *ρ* ≈ 0.45; (**g**) radial profiles of Hurst parameters during the nonlocal phase without (red) and with (black) the NTM.

**Figure 4:**
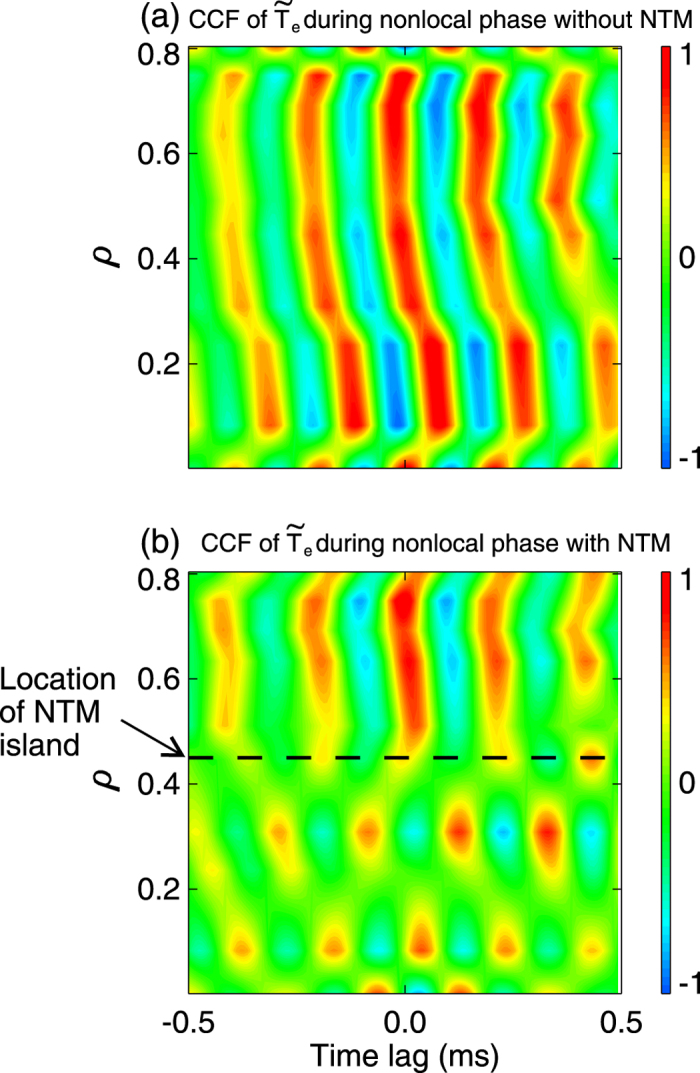
Contour-plots of CCF calculated in filtered ECE signals (4–7 kHz) during the nonlocality (**a**) without and (**b**) with the NTM (#19906)).

Figure 2(a–c) show typical waveforms for a discharge with “nonlocal” transport induced by the SMBI (see vertical yellow bar) in ECRH-heated plasmas. In Fig. 2(d), the time evolution of density fluctuation power is plotted. The ECRH power applied is constant (*P*_*ECRH*_ ≈ 1.32 MW) until switch-off at *t* ≈ 915 ms. After the molecular beam is injected at *t* ≈ 838 ms, the plasma density slightly increases, due to fueling. From the ECE signals plotted in Fig. 2(b), one can clearly see that after the SMBI, the edge *T*_*e*_ (blue curves) drops because of local cooling by the SMBI, but in the core region the electron temperature (red curves) increases unexpectedly. The possible impact by the enhanced ECRH efficiency on the core *T*_*e*_ rise has been eliminated as the core plasma density is not dipped after the SMBI. This transient, long-distance and reversed polarity response where the core *T*_*e*_ rise on response to edge cold pulses is the so-called “nonlocal” heat transport, a phenomenon widely observed in many fusion devices^1^,^2^,^3^,^4^,^5^,^6^,^7^. However, the physical mechanisms responsible for triggering the nonlocal transport remain unclear. Code simulations on TEXT^24^ and TFTR^25^ as well as our modelling indicate that the nonlocal effects cannot be reproduced with transport coefficients that are functions only of local thermodynamic variables. At present, several theoretical models have been proposed^8^,^9^,^10^,^11^,^12^ to explain the nonlocal effect. Among them the avalanche paradigm (related to self-organized criticality) attempts to unravel the transport enigma via the interplay between individual turbulent eddies and large-scale transport events^26^,^27^. Here, several localized eddies cooperate to produce a transient, extended transport events, or avalanche. Avalanches can propagate outward (as local excesses) or inward (as voids). In HL-2A, experimental evidence indicates that this avalanche-like transport may play a crucial role in the distant nonlocal transport events. Typical avalanche features, such as self-similarity and long-range radial correlations in temperature fluctuations (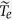), are depicted in Figs 3 and 4, for a similar nonlocal shot. In Fig. 3(f), the red curves, calculated by the structure function (SF), and rescaled range (R/S) analyses of the 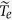 data measured at *ρ* ≈ 0.45, show a high slope, i. e., the Hurst exponent (*H* ≈ 0.78) during the first nonlocal phase (before the NTM). Here, the *q*th-order SF is defined as the *q*th moment of the increments of *X*(*t*) for a time series of *X* ≡ {*X*_*t*_: *t* = 1, 2,..., *n*}, i. e., *S*_*x*,*q*_(*τ*) ≡ 〈|*X*(*t* + *τ*) − *X*(*t*)|^*q*^〉 = *cτ*^*qH*^ 28; and the R/S ratio is defined as the maximal range of the integrated signal normalized by the standard deviation, i. e., *R*(*n*)/*S*(*n*) = [max(0, *W*_1_, *W*_2_, …, *W*_*n*_) − min(0, *W*_1_, *W*_2_, …, *W*_*n*_)]/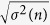 and it depends asymptotically on *n* by *R*(*n*)/*S*(*n*)
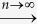
*cn*^*H*^, where *W*_*k*_ = 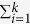
*X*_*i*_ − 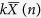, *c* is approximately constant and *H* is the Hurst exponent^29^, which equals to 0.5 for a random process and is larger than 0.5 for a sequence with long-term correlations. The radial profile of the Hurst parameter during that period further indicates that the *H* values are close to 0.8 at all radii (see red points in Fig. 3(g)), suggesting large diffusive propagation. Meanwhile, the cross-correlation function (CCF) of 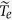 between multi-channel ECE signals exhibits radially long-distance inward propagation in temperature fluctuations (see Fig. 4(a)). All these features are consistent with the avalanche picture. More details on the avalanche characteristics have been described in ref. 30.

In Fig. 2, we notice that along with the nonlocal response in *T*_*e*_, an NTM is excited at *t* ≈ 844 ms. The mode numbers have been identified as *m*/*n* = 3/2 with a frequency *f* ≈ 7 kHz, as seen in the frequency spectrum of the SXR signal in Fig. 2(c). The neoclassical nature of the tearing modes is illustrated in Fig. 2(e), where the variation in the fluctuation amplitude of the radial magnetic field (square root of 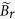 ∝ island width) around the mode frequency (*f* = 6–9 kHz) shows approximately a linear relation with *β*_*N*_[ = *β*(100*aB*/*I*_*p*_)] in five shots having the 3/2 islands. Another character of the NTM can also be seen in Fig. 2(a,c). After the ECRH is switched off at *t* ≈ 915 ms, the saturated island width quickly drops on a time scale of ~10 ms, much shorter than the current diffusion time (*μ*_0_*a*^2^/*η* ~ 100 ms) for conventional tearing modes in HL-2A. Similar results have been observed in the TCV tokamak^31^.

**Figure 5:**
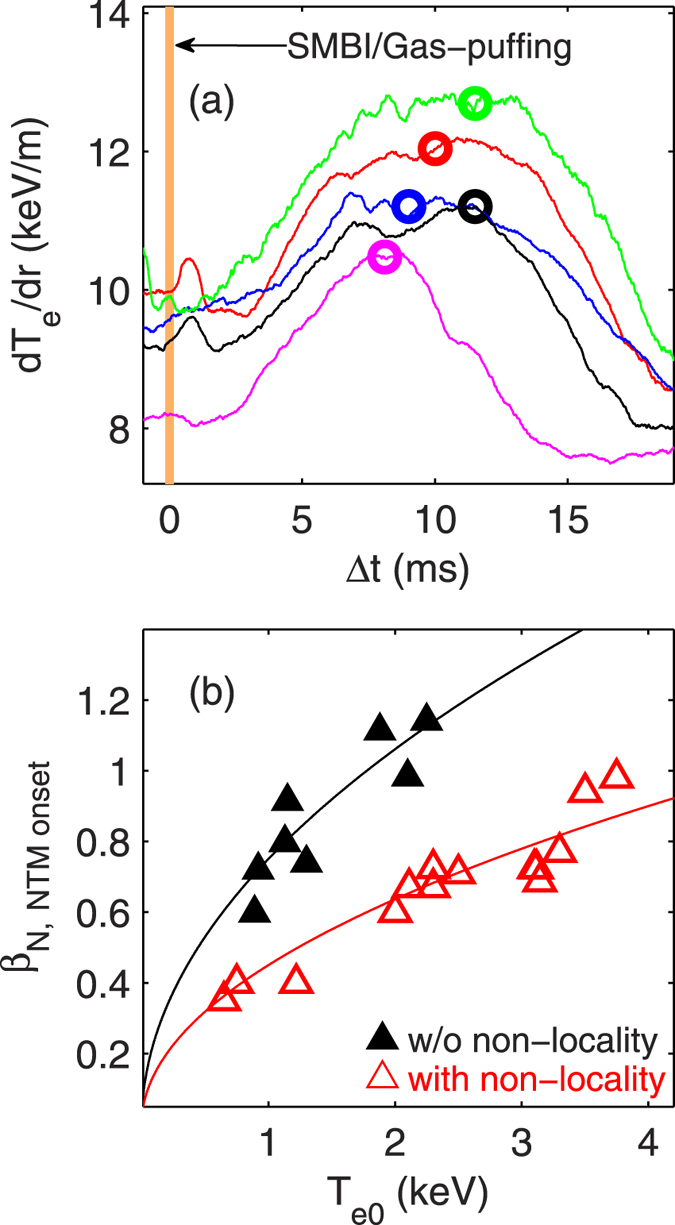
(**a**) Radial gradient of electron temperature (*dT*_*e*_/*dr*) between two radial loci around *q* = 3/2 surface as a function of time delay referred to the SMBI/gas puffing time (Δ*t* = *t* − *t*_*SMBI*/*gas*−*puff*_) across the nonlocal period. The open circles denote the onset times of the 3/2 NTMs in these shots; (**b**) critical values of *β*_*N*_ for the NTM onset with (red) and without (black) nonlocal effects versus the central electron temperature.

For investigating the role of the nonlocality in triggering the NTM, we first inspect the location and onset time of the NTM during the nonlocal transport. In Fig. 2, one can see that between the time of SMBI and the NTM onset (see vertical black dashed line), the *T*_*e*_ increases in the core and decreases in the edge, respectively. At the inversion surface of *ρ* = 0.46, the change in *T*_*e*_ is minimal (≈0). It is found that the *q* = 3/2 rational surface, which is determined from the phase jump in *T*_*e*_ fluctuations measured by ECE, is located around the inversion surface of the nonlocality. Further investigation on other shots shows that the NTM locations are all located close to the inversion surface. In Fig. 5(a), the radial gradient of the local electron temperature, *dT*_*e*_/*dr*, between two radial loci around the inversion surface is drawn versus the time delay relative to the SMBI/gas puffing time (Δ*t* = *t* − *t*_*SMBI*/*gas*−*puff*_), for five “nonlocal” shots. The open circles mark the onset times of the 3/2 NTMs in these discharges. One can see that the NTMs are always triggered when the local temperature gradient reaches its maximum. These results are consistent with theoretical predictions^13^,^15^, which state that a large enough temperature (or pressure) gradient, which is linked to a large bootstrap current, is the crucial element that drives the NTM. Concerning the seeding process of the NTM, prevailing theories presume that the NTM is linearly stable^13^. The development of NTMs requires a seed island whose width must exceed a critical island width, *w*_*crit*_. However, in our experiment no visible MHD mode (e. g., sawtooth activities, fishbones, Alfvénic modes and edge localized modes (ELMs) as seed islands) is observed prior to the NTM, as shown in Fig. 2(c). Although a transient increase is observed in the density fluctuation power during the nonlocal phase (see Fig. 2(d)), there is no clear evidence indicating nonlinear energy coupling between electrostatic and magnetic turbulence via the bicoherence analysis^32^. In our case, there are no obvious ambient seeds. However other possible explanations are that the gradual increase of the temperature (or pressure) gradient before the NTM onset may induce small changes in the current profile and hence minor (invisible) tearing modes, to seed the NTM. Similar “seedless” NTMs have also been reported in other fusion devices^31^,^33^.

In the above experiments, because the NTM might be self-generated with the nonlocality, its onset is mainly attributed to the transient enhancement of the local pressure gradient around the *q* = 3/2 rational surface. Thus, the critical values of *β*_*N*_ to induce the NTMs in nonlocal discharges are substantially lower than those without nonlocal transport, as illustrated in Fig. 5(b). This gives us a serious caution that in fusion devices the control of NTMs could be more challenging than we expect, since strong perturbation of local parameters can trigger NTMs at a relatively low *β*_*N*_. In case of high *β*_*N*_ plasmas with internal transport barriers (ITBs), the local perturbation of the ITB (or ELMs and sawteeth) may also induce the NTM.

In the present study, a significant influence of the NTM on the truncation of the nonlocal responses has been observed. Figure 3(a–e) plots time evolutions of plasma density, multi-channel ECE signals and the contour-plot of the frequency spectrum of the Mirnov signal in a discharge with two nonlocal transport induced by edge gas-puffing. After the first gas-puffing at *t* = 570 ms, the nonlocal event appears, and then the 3/2 NTM is excited with *f* = 6–8 kHz, similar to that depicted in Fig. 2. After the occurrence of the NTM, the second gas-puffing is injected at *t* = 770 ms, which also triggers a nonlocal transport event. However, for the second nonlocality, the changes of *T*_*e*_ in both edge and core regions are much smaller than the first one. These results indicate that the nonlocality is regulated by the self-generated NTM.

**Figure 6:**
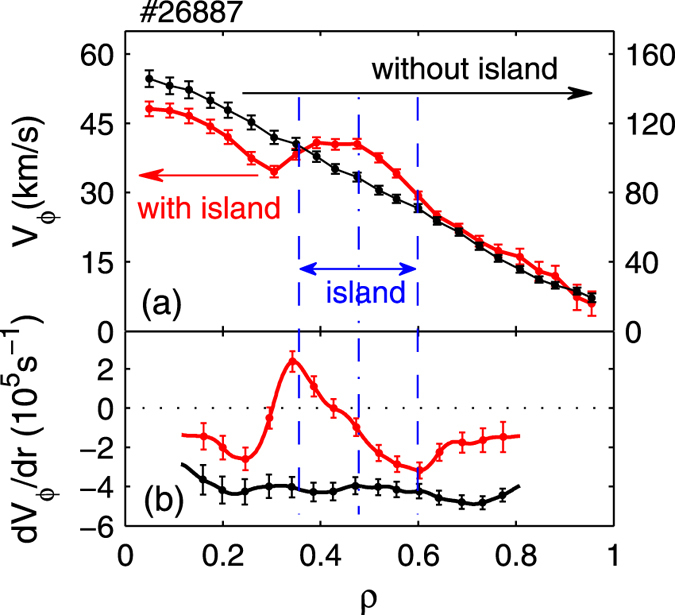
Radial dependence of (**a**) toroidal flow and (**b**) toroidal flow shear without (black) and with (red color) the *m*/*n* = 2/1 magnetic island.

To understand the above phenomena, we have first compared the change of avalanche properties between the two nonlocal periods. In Fig. 3(f,g), the Hurst parameter estimated at *ρ* ≈ 0.45 and the radial dependence of *H* during the second nonlocal phase (with NTM) are plotted (black points) for a comparison with the first nonlocal phase. With NTM, the Hurst exponents drop abruptly in the inner region, suggestive of a return to diffusive transport, whereas in the outer area they are nearly unchanged. The truncation of avalanche-like transport by the NTM can be seen more clearly in Fig. 4(b), where the contour-plot of CCF between ECE signals in the second nonlocal period is drawn. The figure shows that the radial correlation in 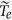 remains large in the outer zone (*ρ* ≈ 0.5–0.8), consistent with high Hurst parameters in that region, as shown in Fig. 3(g). However, in the inner region (*ρ* < 0.5), the radial correlation of transport events is truncated. Note that the 3/2 NTM island is located close to *ρ* ≈ 0.45. To study the impact of the magnetic island on the nonlocality in HL-2A, we have compared the radial dependence of the toroidal flow (*V*_*ϕ*_) and flow shear (*dV*_*ϕ*_/*dr*) measured by the CXRS with (red) and without (black) the conventional magnetic island (*m*/*n* = 2/1). The CXRS monitors the carbon line emission (CVI, n = 8 → 7, 529.06 nm). The results are shown in Fig. 6. Without island, the *V*_*ϕ*_ profile is quite smooth with roughly a constant flow shear rate at all radial locations. With the island (~8.6 cm in width), the magnitude of *V*_*ϕ*_ is generally reduced (see Fig. 6(a)). However, in the latter case, the shear rate of *V*_*ϕ*_ is locally enhanced with dual shear layers developed at the edge of the island, due to probably the influence of the magnetic island on local structures of the toroidal flow. Similar results have been observed in LHD, where a poloidally sheared flow occurs in the magnetic island when the island width grows to a certain extent^19^. According to theories and modeling^10^,^26^,^27^, such sheared flows should regulate or truncate the avalanche-like transport events. Hence, the mechanism for the nonlocal transport is weakened. This explains the truncation of the nonlocal transport by NTMs.

## Conclusion

In this Letter, we report the first observation on the interplay between nonlocal heat transport and NTMs generated during the transient nonlocal response at the HL-2A tokamak. The nonlocality is induced by edge cold pulses, along with inward propagating avalanche transport events. The enhanced local pressure gradient at the rational surface can trigger the NTM in relatively low *β* plasmas. With NTMs, locally sheared toroidal flows are developed in the magnetic island. As a result, the nonlocal avalanche transport event is truncated by flow shearing. The results provide significant evidence for understanding dynamical interactions between multi-scale turbulence and large mode structures.

## Additional Information

**How to cite this article**: Ji, X. Q. *et al*. On the interplay between neoclassical tearing modes and nonlocal transport in toroidal plasmas. *Sci. Rep.* **6**, 32697; doi: 10.1038/srep32697 (2016).

## Figures and Tables

**Figure 1:**
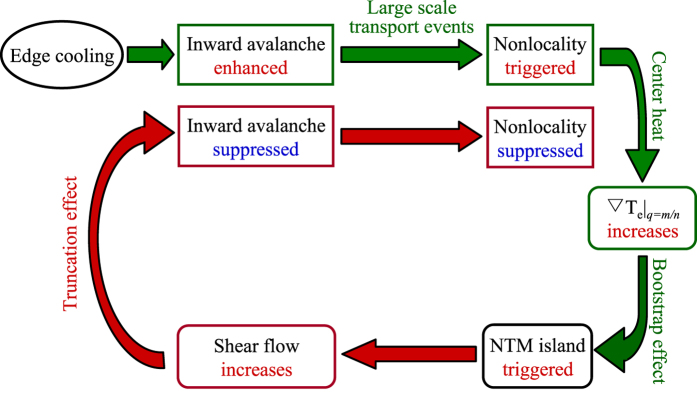
A sketch of the dynamic flow chart for self-regulation of nonlocal transport events by the NTM.

## References

[b1] Kissick M. W. . Transient electron heat diffusivity obtained from trace impurity injection on TFTR. Nucl. Fusion 34, 349–358 (1994).

[b2] Gentle K. W. . Strong nonlocal effects in a tokamak perturbative transport experiment. Phys. Rev. Lett. 74, 3620–3623 (1995). 10058251 10.1103/PhysRevLett.74.3620

[b3] Callen J. D. & Kissick M. W. Evidence and concepts for non-local transport. Plasma Phys. Controlled Fusion 39, B173–B188 (1997).

[b4] Mantica P. . Nonlocal transient transport and thermal barriers in rijnhuizen tokamak project plasmas. Phys. Rev. Lett. 82, 5048–5051 (1999).

[b5] Sun H. J. . Experiment of non-local effect with SMBI on HL-2A. Plasma Phys. Controlled Fusion 52, 045003 (2010).

[b6] Inagaki S. . Fluctuations with long-distance correlation in quasi-stationary and transient plasmas of LHD. Nucl. Fusion 52, 023022 (2012).

[b7] Ida K. . Towards an emerging understanding of non-locality phenomena and non-local transport. Nucl. Fusion 55, 013022 (2015).

[b8] Kubota T. . *Avalanche dynamics of collapse and non-local model of transport,* The 24th European Physical Society Conference on Controlled Fusion and Plasma Physics P4.099 (Berchtesgaden, Germany, June 9–13, 1997).

[b9] Parail V. V. . Transport analysis of transient phenomena in JET. Nucl. Fusion 37, 481–492 (1997).

[b10] Diamond P. H. & Hahm T. S. On the dynamics of turbulent transport near marginal stability. Phys. Plasmas 2, 3640-3649 (1995).

[b11] Wang Z. H., Diamond P. H., Gürcan Ö. D., Garbet X. & Wang X. G. Turbulence propagation in heat flux-driven plasmas: implications for temperature profile structure.Nucl. Fusion 51, 073009 (2011).

[b12] Gürcan Ö. D. & Diamond P. H. Zonal flows and pattern formation. J. Phys. A: Math. Theor. 48, 293001 (2015).

[b13] Wilson H. R. . The collisionality dependence of tokamak *β*-limits. Plasma Phys. Controlled Fusion 38, A149–A163 (1996).

[b14] Chang Z. . Observation of nonlinear neoclassical pressure-gradientdriven tearing modes in TFTR. Phys. Rev. Lett. 74, 4663–4666 (1995). 10058567 10.1103/PhysRevLett.74.4663

[b15] Sauter O. . Beta limits in long-pulse tokamak discharges. Phys. Plasmas 4, 1654–1664 (1997).

[b16] PoliE., PeetersA. G., BergmannA., GünterS. & PinchesS. D. Reduction of the ion drive and 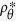 scaling of the neoclassical tearing mode.Phys. Rev. Lett. 88, 075001 (2002).1186390410.1103/PhysRevLett.88.075001

[b17] Goodman T. P., Felici F., Sauter O. & Graves J. P. Sawtooth pacing by real-time auxiliary power control in a tokamak plasma. Phys. Rev. Lett. 106, 245002 (2011). 21770577 10.1103/PhysRevLett.106.245002

[b18] Gerhardt S. P., Anderson D. T. & Talmadge J. N. Calculations of neoclassical viscous damping on flux surfaces near magnetic islands in the helically symmetric experiment. Phys. Plasmas 12, 012504 (2005).

[b19] Ida K. . Observation of plasma flow at the magnetic island in the large helical device. Phys. Rev. Lett. 88, 015002 (2002). 11800959 10.1103/PhysRevLett.88.015002

[b20] Estrada T. . Sheared flows and transition to improved confinement regime in the TJ-II stellarator. Plasma Phys. Controlled Fusion 51, 124015 (2009).

[b21] Duan X. R. . Overview of experimental results on HL-2A. Nucl. Fusion 49, 104012 (2009).

[b22] Shi Z. B. . Observations of the pulse perturbation during multi-pulse molecular beam injection on HL-2A. Plasma Phys. Controlled Fusion 47, 2019–2028 (2005).

[b23] Wei Y. L. . High spatial and temporal resolution charge exchange recombination spectroscopy on the HL-2A tokamak. Rev. Sci. Instrum. 85, 103503 (2014). 25362389 10.1063/1.4897186

[b24] Gentle K. W. . An experimental counter-example to the local transport paradigm. Phys. Plasmas 2, 2292–2298 (1995).

[b25] Kissick M. W., Callen J. D., Fredrickson E. D., Janos A. C. & Taylor G. Non-local component of electron heat transport in TFTR. Nucl. Fusion 36, 1691–1701 (1996).

[b26] Newman D. E., Carreras B. A., Diamond P. H. & Hahm T. S. The dynamics of marginality and self-organized criticality as a paradigm for turbulent transport. Phys. Plasmas 3, 1858–1866 (1996).

[b27] Carreas B. A., Newman D., Lynch V. E. & Diamond P. H. A model realization of self-organized criticality for plasma confinement. Phys. Plasmas 3, 2903–2911 (1996).

[b28] Davis A., Marshak A., Wiscombe W. & Cahalan R. Multifractal characterizations of nonstationarity and intermittency in geophysical fields: observed, retrieved, or simulated. J. Geophys. Res. 99, 8055–8072 (1994).

[b29] Hurst H. E. Long-term storage capacity of reservoirs. Trans. Am. Soc. Civ. Eng. 116, 770–808 (1951).

[b30] Pan O. . Evidence of enhanced self-organized criticality (SOC) dynamics during the radially non-local transient transport in the HL-2A tokamak. Nucl. Fusion 55, 113010 (2015).

[b31] Reimerdes H., Sauter O., Goodman T. & Pochelon A. From current-driven to neoclassically driven tearing modes. Phys. Rev. Lett. 88, 105005 (2002). 11909366 10.1103/PhysRevLett.88.105005

[b32] Ji X. . *Interaction between neoclassical tearing modes and non-local transport in HL-2A,* The 25th IAEA Fusion Energy Conference EX/6-4 (Saint Petersburg, Russia, October 13–18, 2014).

[b33] Gude A., Günter S., Sesnic S. & ASDEX Upgrade Team. Seed island of neoclassical tearing modes at ASDEX Upgrade. Nucl. Fusion 39, 127–131 (1999).

